# Upper instrumented vertebra–femoral angle and correlation with proximal junctional kyphosis in adult spinal deformity

**DOI:** 10.1007/s43390-021-00408-1

**Published:** 2021-09-03

**Authors:** Hao-Hua Wu, Dean Chou, Kevork Hindoyan, Jeremy Guinn, Joshua Rivera, Pingguo Duan, Minghao Wang, Zhuo Xi, Bo Li, Andrew Lee, Shane Burch, Praveen Mummaneni, Sigurd Berven

**Affiliations:** 1 Department of Orthopaedic Surgery, University of California, San Francisco, 500 Parnassus Avenue, MU 320-W, San Francisco, CA 94143 USA; 2 Department of Neurological Surgery, University of California, San Francisco, San Francisco, USA

**Keywords:** Proximal junctional kyphosis, Adult spinal deformity, Surgical complication

## Abstract

**Introduction:**

Although matching lumbar lordosis (LL) with pelvic incidence (PI) is an important surgical goal for adult spinal deformity (ASD), there is concern that overcorrection may lead to proximal junctional kyphosis (PJK). We introduce the upper instrumented vertebra–femoral angle (UIVFA) as a measure of appropriate postoperative position in the setting of lower thoracic to pelvis surgical correction for patients with sagittal imbalance. We hypothesize that a more posterior UIV position in relation to the center of the femoral head is associated with an increased risk of PJK given compensatory hyperkyphosis above the UIV.

**Methods:**

In this retrospective cohort study, adult patients undergoing lower thoracic (T9–T12) to pelvis correction of ASD with a minimum of 2-year follow-up were included. UIVFA was measured as the angle subtended by a line from the UIV centroid to the femoral head center to the vertical axis. Patients who developed PJK and those who did not were compared with preoperative and postoperative UIVFA as well as change between postoperative and preoperative UIVFA (deltaUIVFA).

**Results:**

Of 119 patients included with an average 3.6-year follow-up, 51 (42.9%) had PJK and 24 (20.2%) had PJF. Patients with PJK had significantly higher postoperative UIVFA (12.6 ± 4.8° vs. 9.4 ± 6.6°, *p* = 0.04), deltaUIVFA (6.1 ± 7.6° vs. 2.1 ± 5.6°, *p* < 0.01), postoperative pelvic tilt (27.3 ± 9.2 vs. 23.3 ± 11, *p* = 0.04), postoperative lumbar lordosis (47.7 ± 13.9° vs. 42.4 ± 13.1, *p* = 0.04) and postoperative thoracic kyphosis (44.9 ± 13.2 vs. 31.6 ± 18.8) than patients without PJK. With multivariate logistic regression, postoperative UIVFA and deltaUIVFA were found to be independent risk factors for PJK (*p* < 0.05). DeltaUIVFA was found to be an independent risk factor for PJF (*p* < 0.05). A receiver operating characteristic (ROC) curve for UIVFA as a predictor for PJK was established with an area under the curve of 0.67 (95% CI 0.59–0.76). Per the Youden index, the optimal UIVFA cut-off value is 11.5 degrees.

**Conclusion:**

The more posterior the UIV is from the femoral head center after lower thoracic to pelvis surgical correction for ASD, the more patients are at risk for PJK. The greater the magnitude of posterior translation of the UIV from the femoral head center from preop to postop, the greater the likelihood for PJF.

## Introduction

Proximal junctional kyphosis (PJK) is a notable complication after adult spinal deformity that most commonly occurs at a rate of 20–40% [1]. The proximal junctional angle (PJA) is the cobb angle between the caudal endplate of the upper instrumented vertebra (UIV) and the cephalad endplate of the vertebra two levels above the UIV (UIV + 2), and patients with PJK have a PJA ≥ 10° or at least 10° greater than the preoperative PJA measurement [2, 3]. Clinical presentation of PJK ranges from asymptomatic to increased pain to functional or neurological deficits that require revision surgery, defined as proximal junctional failure (PJF) [1, 4–6].

Given the clinical ramifications of PJK, many studies have focused on modifiable risk factors, including radiographic parameters that could inform preoperative planning [3, 5–7]. In particular, overcorrection in the sagittal plane has been correlated with PJK [5, 7]. For example, Maruo et al. suggests that overcorrection of lumbar lordosis (LL) > 30 degrees from preoperative measurement could be a risk factor for PJK [7]. However, recent literature suggests measuring lumbar lordosis itself is not an accurate proxy for predicting PJK in surgical constructs extending into the thoracic spine [8, 9]. Therefore, there remains a need for a radiographic measurement that quantifies overcorrection in long instrumented fusion.

**Fig. 1:**
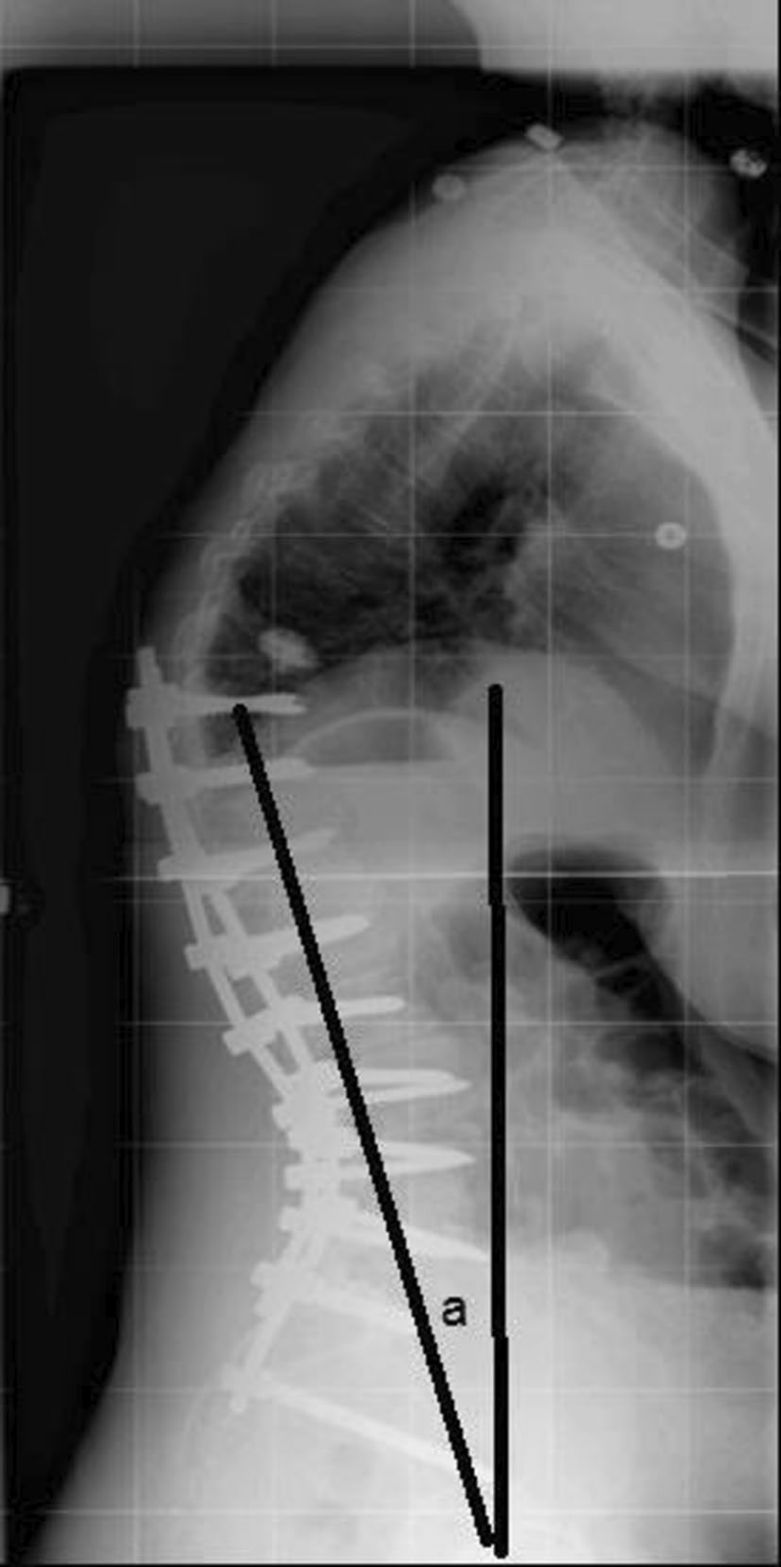
The upper instrumented vertebra–femoral angle (UIVPA) is represented by “a” as the angle subtended by a line from the UIV centroid to the femoral head center to a vertical reference line

In this study, we introduce the upper instrumented vertebra–femoral angle (UIVFA) as a measure of appropriate postoperative position of the UIV in the setting of a lower thoracic to sacrum surgical correction for adult spinal deformity (ASD) patients. The UIVFA is the angle subtended by a line from UIV centroid to the center of the femoral heads to a vertical reference line (Fig. 1). The goal of this angle is to inform how far posterior the UIV is from the center of the femoral head as a proxy of overcorrection.

The purpose of this study was to evaluate utility of the UIVFA in the measurement of ASD, assess its correlation with other radiographic parameters, and assess correlation of the rates of PJK after lower thoracic (T9–T12) to sacrum ASD correction. We hypothesize that a more posterior UIV position in relation to the center of the femoral head is associated with an increased risk of PJK given compensatory hyperkyphosis above the UIV.

## Methods

In this single-center retrospective cohort study, adult patients undergoing lower thoracic (T9–T12) to sacrum open posterior fusion by four board-certified spine surgeons were included. Inclusion criteria were minimum 2 years of follow-up, ASD surgery from lower thoracic to sacrum, preoperative and postoperative long cassette standing radiographs to evaluate spinopelvic parameters, and clinical follow-up. Patients with malignancy, infection, traumatic injury, or less than 2 years of follow-up were excluded. All patients were evaluated with a 36-inch standing anteroposterior (AP) and lateral spine radiographs preoperatively, postoperatively at 1 month, 3 months, 6 months, 1 year, 2 years, and at final follow-up.

Demographic factors, such as age, gender and number of levels instrumented, were collected. The upper instrumented vertebra–femoral angle (UIVFA) is determined by a line drawn from the UIV centroid down to the center of the femoral heads subtended by vertical reference line. The greater the angle, the farther posterior the UIV is from the center of the femoral heads. UIV posterior to the femoral head center was positive (+) and UIV anterior the femoral head center was negative (−). UIVFA values from different lower thoracic UIV levels (e.g., T9, T10, T11, T12) were compared. DeltaUIVFA is determined as the difference between the postoperative and preoperative UIVFA; the greater the difference, the greater the degree of overcorrection. Preoperative and postoperative UIVFA, pelvic tilt (PT), sacral slope (SS), pelvic incidence (PI), lumbar lordosis (LL), PI–LL, T1 pelvic angle (T1PA), thoracic kyphosis (TK) measured from T5 to T12, sagittal vertical axis (SVA), and central sacral vertical line (CSVL) were measured. The CSVL was measured as the distance between the vertical line drawn perpendicular to the floor from the geometric center of S1 to C7 plumbline. The proximal junctional angle (PJA) was defined as the sagittal Cobb angle measurement between the inferior endplate of the upper instrumented vertebrae (UIV) and the superior endplate of 2 vertebrae above (UIV + 2). Proximal junctional kyphosis was defined as a PJA of ≥ 10° or ≥ 10°compared to the preoperative PJA. Complications were also recorded. Proximal junctional failure (PJF) was defined as any PJK patient that required revision surgery. Patients with PJK were compared to those without PJK.

### Data analysis

Categorical variables were analyzed with chi-square while continuous variables were compared with standard *t* test. Pearson’s correlation was used to quantify the correlation between UIVPA and other spinopelvic parameters. Univariate analysis of preoperative and postoperative variables was performed with a Student* t* test. Significant variables were then analyzed with multivariate binary logistic regression analysis. A receiver operating characteristic (ROC) curve was analyzed and the area under the curve (AUC) was calculated for UIVFA. The cut-off value for UIVFA was also identified using the Youden index. Statistical analysis was carried out using Stata (StataCorp LLC, College Station, TX). An alpha of < 0.05 was used to determine significance.

## Results

There were 119 patients who met the inclusion criteria out of 289 patients screened from 2011 to 2017, with a mean follow-up of 3.6 years (Range; 2–11 years). The mean age of all patients was 62.9 ± 10, and 81 (68%) were females. Overall, the average preoperative SVA was 89.5 mm, preoperative PT was 27.1°, and preoperative PI–LL mismatch was 27.0°. These values improved postoperatively to an average SVA of 56.8 mm, average PT of 24.7°, and average PI–LL of 11.1°. There were no significant differences in UIVFA measurements from different lower thoracic UIV levels. The mean preoperative UIVFA was 7.0 ± 0.7°, which changed postoperatively to 10.8 ± 0.6°. The mean deltaUIVFA was 3.8 ± 0.6°. Fifty-one patients (42.9%) were found to have PJK. Of those patients, 24 (20.2%) went on to have revision surgery and were categorized in the PJF group.

There was no difference in age and sex between patients with and without PJK (Table 1). Of the preoperative radiographic parameters, both groups had no significant differences with respect to SS, PT, PI, LL, PI–LL, TK (T5–T12), CSVL and UIVFA. Patients with PJK had significantly higher preoperative T1PA (30.8 ± 11.9° vs. 25.3 ± 9.7°, *p* = 0.013), and higher SVA (103.1 ± 67 vs. 79.2 ± 54, *p* = 0.034) compared to patients without PJK. There were no significant preoperative radiographic differences between patients with and without PJF.

**Table 1:** Demographics and preoperative radiographic measurements of patients with and without PJK

	PJK (*n* = 51)	No PJK (*n* = 68)	*p* value
Age	64.6 ± 7.1	61.6 ± 12.4	0.16
Female (%)	37 (72%)	44 (65%)	0.36
Preop SS	30.6 ± 9.8	31.1 ± 12.9	0.83
Preop PT	28.8 ± 8.2	25.7 ± 10.3	0.08
Preop PI	59.5 ± 11.4	56.4 ± 13.6	0.19
Preop LL	29.0 ± 17.8	32.8 ± 19.3	0.28
Preop PI–LL	30.0 ± 17.7	24.6 ± 19.3	0.11
Preop T1PA	30.8 ± 11.9	25.3 ± 9.7	**0.013**
Preop TK (T5–T12)	24.6 ± 15.0	25.1 ± 21.4	0.89
Preop SVA (mm)	103.1 ± 67	79.2 ± 54	**0.034**
Preop CSVL (mm)	21.1 ± 27	27.0 ± 42	0.42
Preop UIVFA	6.5 ± 8.8	7.3 ± 7.2	0.58

Bolded text means a significant *p*-value (e.g. *p* < 0.05)

One-month postoperatively, first standing radiographs showed patients with and without PJK had similar SS, LL, PI–LL, SVA, CSVL (Table 2). Patients with PJK had significantly higher postoperative PT (27.3 ± 9.2° vs. 23.3 ± 11.0°, *p* = 0.04), postoperative LL (47.7 ± 13.9 vs. 42.4 ± 13.1, *p* = 0.036), postoperative thoracic kyphosis at T5-T12 (44.9 ± 13.2° vs. 31.6 ± 18.8°, *p* < 0.01), postoperative UIVFA (12.6 ± 4.8° vs. 9.4 ± 6.6°, *p* = 0.041) and change of UIVFA from postop to preop (deltaUIVFA, 6.1 ± 7.6 vs. 2.1 ± 5.6, *p* < 0.01) compared to patients without PJK. Patients with PJF had significantly increased deltaUIVFA (7.9 ± 6.2 vs. 2.8 ± 6.6, *p* < 0.01), postoperative PT (29.6 ± 8.8° vs. 23.9 ± 10.5°, *p* = 0.02) and postoperative thoracic kyphosis at T5–T12 (47.4 ± 16° vs. 34.9 ± 17.4°, *p* < 0.01). Patients with and without PJF had no significant difference with postoperative UIVFA (12.8 ± 5.6 vs. 10.3 ± 6.2, *p* = 0.07).

**Table 2:** Postoperative radiographic measurements of patients with and without PJK

	PJK (*n* = 51)	No PJK (*n* = 68)	*p* value
Postop SS	32.7 ± 9.5	32.5 ± 10.3	0.90
Postop PT	27.3 ± 9.2	23.3 ± 11.0	**0.04**
Postop LL	47.7 ± 13.9	42.4 ± 13.1	**0.04**
Postop PI–LL	10.1 ± 11.7	11.7 ± 16.3	0.59
Postop T1PA	24.6 ± 8.9	23.6 ± 12.0	0.62
Postop TK (T5–T12)	44.9 ± 13.2	31.6 ± 18.8	** < 0.01**
Postop SVA (mm)	57.3 ± 39.3	56.4 ± 48.9	0.92
Postop CSVL (mm)	17.2 ± 14.1	19.1 ± 24.5	0.63
Postop UIVFA	12.6 ± 4.8	9.4 ± 6.6	**0.04**
DeltaUIVFA	6.1 ± 7.6	2.1 ± 5.6	** < 0.01**

Bolded text means a significant p-value (e.g. *p* < 0.05)

**Fig. 2:**
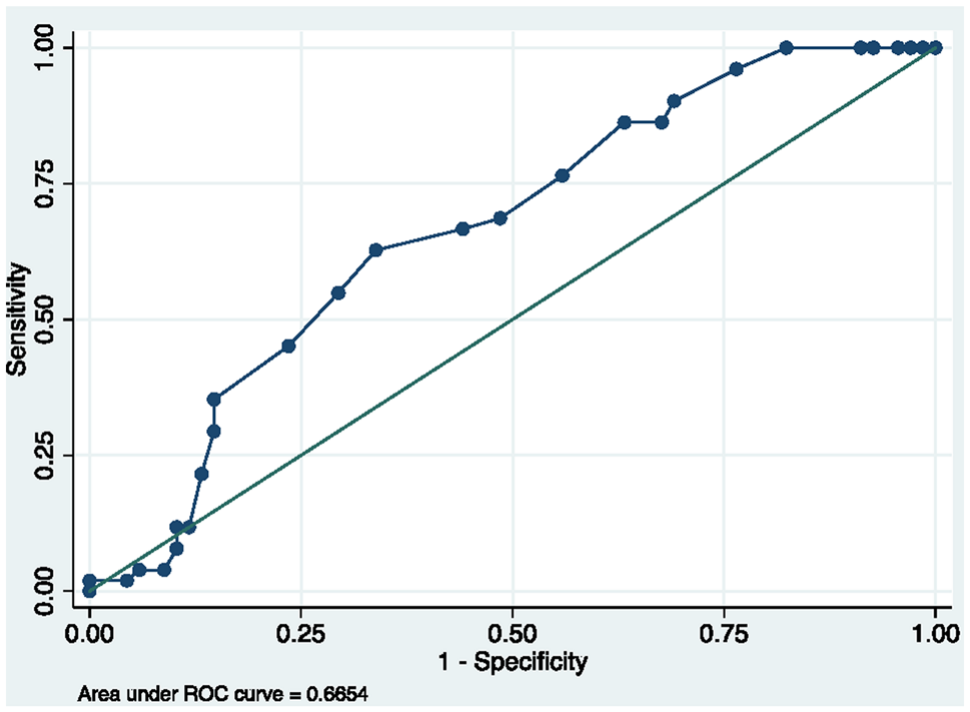
Receiver operating characteristic (ROC) curve for UIVFA as a predictor for PJK

**Fig. 3:**
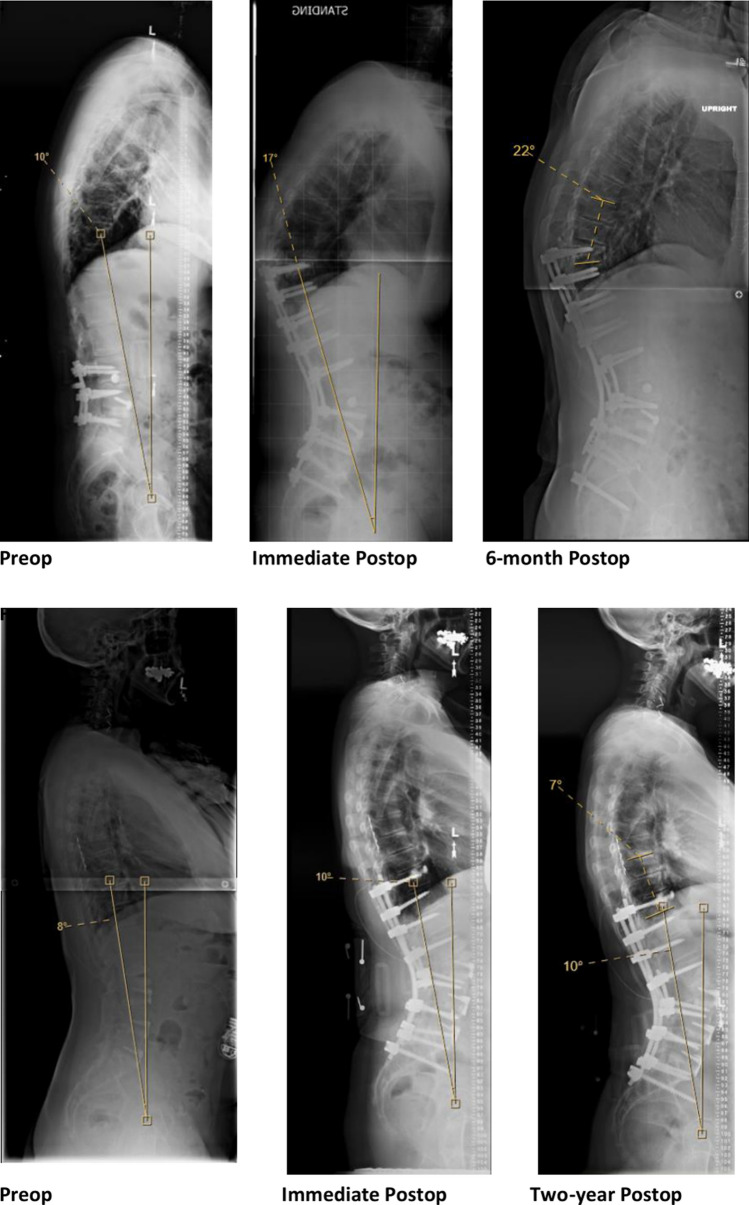
Comparison of UIVFA between patient with and without PJK at 2-year follow-up. **a** UIVFA of PJK patient at preop, immediate postop and 6-month postop. At immediate postop, patient’s UIVFA is 17° which is greater than the optimal 11.5° cut-off. **b** UIVFA of patient without PJK at preop, immediate postop and 2-year postop. At immediate postop, patient’s UIVFA is 10° which meets the optimal 11.5° cut-off

Multivariate logistic regression analysis showed postoperative UIVFA [beta 0.06 (95% CI 0.023–0.10, *p* = 0.02)] and deltaUIVFA [beta 0.078 (95% CI 0.01–0.17, *p* = 0.04)] to be independent risk factors associated with PJK. Neither preoperative SVA nor postoperative LL was found to be independent risk factors. A receiver operating characteristic (ROC) curve for UIVFA as a predictor for PJK was established with an area under the curve of 0.67 (95% CI: 0.59–0.76) (Fig. 2). As per the Youden index, the optimal postoperative UIVFA cut-off value is 11.5° (sensitivity 0.63, specificity 0.66) indicating that a postoperative UIVFA greater than 11.5° in low thoracic to sacrum posterior fusion may be a risk factor for PJK (Fig. 3).

## Discussion

To restore sagittal balance, it is important to align the center of the cranial mass with the center of the femoral heads and lower extremities [10]. However, many factors can lead to overcorrection, including increase of lordosis in the prone position when only a standing film is used as a preoperative guide, differences in normal SVA and PI–LL mismatch based on age group, and traditional alignment goals that are not patient centric (e.g., SVA < 5 cm, PT < 20, PI–LL < 10) [11–13]. Lafage et al., for instance, found that patients ≥ 75 years old had a normal mean SVA of 78.1 cm and a normal PI–LL mismatch of 16.7, which predisposes to overcorrection if not taken into account [11, 12]. Our data show that overcorrection of alignment, including the final position of the UIV in relation to the femoral head and the absolute change in UIVFA from preop to postop, is an independent risk factor for development of PJK in patients undergoing LT to sacrum surgical correction of ASD. In the operating room, UIVFA can be obtained with a PACS or goniometer measurement from a lateral radiograph that captures both the UIV and femoral heads. Ideally, this image would be obtained after the completion of surgical corrective techniques, and UIVFA could be used as an additional data point in determining adequacy of correction. The optimal cut-off point of UIVFA to avoid overcorrection and risk of PJK is 11.5°, so anything below this value is preferred.

The results of our study are similar to literature that suggests overcorrection of sagittal alignment can lead to PJK. Line et al., for instance, found that patients who were aligned based on age-appropriate PI–LL mismatch were significantly less likely to have PJK requiring revision surgery [14]. Lafage et al. also found that patients with greater correction as determined by SVA and PI–LL mismatch were more likely to have PJK than those who were appropriately aligned [12]. Similarly, our data shows that higher overcorrection as determined by UIV from center of femoral heads as well as absolute change of UIVFA is correlated to PJK. Although the etiology of PJK is unclear, it is likely that patients who are overcorrected compensate by hyperkyphosing above the fused UIV.

Unlike other literature, our study is the first to our knowledge to incorporate the UIV into a radiographic measurement concerning sagittal balance. The UIVFA offers three advantages. First, the UIV is the transition point between the rigid and mobile spine, and accounting for this transition point can help serve as a more accurate proxy for overcorrection. Second, UIVFA is feasible and practical to measure intraoperatively. Traditionally used radiographic measurements such as SVA, T1PA or global tilt require a full-length film and are more tedious to ascertain [15, 16]. Third, prior measures of overcorrection used in prior studies, such as age-matched PI–LL mismatch, PT and lumbar lordosis, do not take into account different anatomic morphologies, such as Roussouly type [9, 17]. UIVFA, on the other hand, serves as a patient-centric indicator of how far posterior the UIV is from ideal alignment.

There are several limitations to this study. First, this study was retrospective. Second, due to the retrospective nature of the study, we were not able to collect and correlate patient-reported outcome measures (PROMs), such as the Oswestry Disability Index, to UIVFA. However, previous literature has assessed the correlation between PROMs and PJK, which a high UIVFA is correlated with. Third, given the follow-up requirements and that patients were only recruited from a single institution, the number of subjects in our study are not as high as comparable multicenter studies. Therefore, our study may have been underpowered to show differences in events that occurred with less frequency, such as PJF. This may explain why UIVFA and deltaUIVFA were not found to be independent risk factors for patients with PJF, although they were found to be independent risk factors for PJK, which is a more frequent outcome variable. Thus, future multicenter, prospective studies on this topic are warranted. Fourth, the surgical corrections for these patients were heterogenous among the four surgeons, and patients with Smith-Peterson osteotomies (SPO) alone, pedicle-subtraction osteotomies (PSO), vertebral column resection (VCR) or a combination of these osteotomies were compared to one another. However, the consistency of these data is that all patients had ASD correction with lower thoracic to sacrum long-segment fusions, and hopefully this can make the results of this study more generalizable.

## Conclusion

Our study found a 42.9% rate of PJK and a 20.2% rate of PJF, which is comparable to what is described in the literature. Patients with higher postoperative UIVFA and deltaUIVFA, which served as a proxy for overcorrection, were more likely to present with PJK at a minimum of 2-year follow-up. An optimal postoperative UIVFA cut-off of 11.5° is suggested for the prevention of PJK. Further, prospective studies are warranted to further evaluate increased postoperative UIVFA as a proxy for overcorrection and as a risk factor for PJK.
